# Augmenting Paraphrase Generation with Syntax Information Using Graph Convolutional Networks

**DOI:** 10.3390/e23050566

**Published:** 2021-05-02

**Authors:** Xiaoqiang Chi, Yang Xiang

**Affiliations:** College of Electronics and Information Engineering, Tongji University, Shanghai 201804, China; chixiaoqiang@tongji.edu.cn

**Keywords:** paraphrase generation, syntax information, graph convolutional network, sequence-to-sequence

## Abstract

Paraphrase generation is an important yet challenging task in natural language processing. Neural network-based approaches have achieved remarkable success in sequence-to-sequence learning. Previous paraphrase generation work generally ignores syntactic information regardless of its availability, with the assumption that neural nets could learn such linguistic knowledge implicitly. In this work, we make an endeavor to probe into the efficacy of explicit syntactic information for the task of paraphrase generation. Syntactic information can appear in the form of dependency trees, which could be easily acquired from off-the-shelf syntactic parsers. Such tree structures could be conveniently encoded via graph convolutional networks to obtain more meaningful sentence representations, which could improve generated paraphrases. Through extensive experiments on four paraphrase datasets with different sizes and genres, we demonstrate the utility of syntactic information in neural paraphrase generation under the framework of sequence-to-sequence modeling. Specifically, our graph convolutional network-enhanced models consistently outperform their syntax-agnostic counterparts using multiple evaluation metrics.

## 1. Introduction

Paraphrase generation is the task of restating a sentence with different wording while keeping the same semantic meaning. Since variation is a characteristic of language, paraphrase generation systems could be a key component in numerous natural language processing tasks [1,2]. For instance, paraphrase generation could be used to expand patterns in information extraction systems [3], reformulate queries for information retrieval [4,5], and generate diverse text in question answering [6] and dialog systems. It also finds applications in semantic parsing [7], summarization [8] and sentence simplification [9,10]. More generally, it could be employed as a data augmentation technique to improve the robustness of natural language processing (NLP) models against language variation. Recent years have witnessed great successes in neural machine translation (NMT) and related generation tasks that could be formulated under the sequence-to-sequence learning framework. As a result, the field of paraphrase generation is also gaining attention.

Previous work employs the sequence-to-sequence (seq2seq) model for the task of paraphrase generation [11], where the authors stacked multiple long short-term memory (LSTM) layers with residual connections. It is the first piece of work to explore neural network-based models for paraphrase generation. One highlight of their model is the introduction of residual connections between LSTM layers, which is crucial for efficient training of deep networks, as already demonstrated in the vision community. Their model outperformed multiple seq2seq baselines on three machine translation-oriented evaluation metrics and a sentence similarity metric. Some adopt more complex architectures: Cao et al. [12] assumed that there are two underlying writing modes in real-world paraphrase datasets, and model each mode with a separate decoder. To choose between these two decoders, they conceived a mode predictor, which is formulated as a binary sequence labeling task. Their model outperformed previous approaches in terms of both informativeness and language quality on two tasks: one-sentence abstractive summarization and text simplification. However, they did not experiment with paraphrase datasets. Another line of research resorts to NMT [13,14], where a source sentence is first translated into a pivot language, then the translated sentence is back-translated into the same language as the source sentence. One advantage of these methods is the availability of large-scale bilingual corpora which, they could leverage. Mallinson et al. [13] reported performance gains over phrase-based translation approaches, while Wieting and Gimpel [14] showed the efficacy of their generated paraphrases in learning sentence representations for measuring textual similarity.

None of the above approaches take linguistic knowledge into consideration. The authors of [15,16] utilized lexical resources in the form of synonym dictionaries. Huang et al. [15] made use of information from an off-the-shelf dictionary. Specifically, they extracted appropriate word-level and phrase-level paraphrase pairs from the PPDB database while taking the original sentence into account. Instead of naively replacing words in the source sentence with their counterpart in the paraphrase pairs, they used these paraphrase pairs to construct edit vectors. Edit vectors are responsible for deletion and insertion operations in paraphrase generation. They achieved stronger performance over several baselines. Lin et al. [16] also employed an off-the-shelf dictionary containing synonyms. In addition to using these synonym pairs to guide the decision on whether to generate a new word or replace it with a synonym as in [15], they integrated information of word location with a positional encoding layer in Transformer [17]. An interesting part of their work is that they formulated the locating of synonym candidates as a synonym labeling task, which is trained independently in an early stage. Then, the synonym labeler and the paraphrase generator are jointly trained to perform multi-task learning, where the two tasks share a common encoder. They reported better performance over previous work on both English and Chinese paraphrase datasets.

Wang et al. [18] used semantic information, which is represented as PropBank [19] style frame-semantic labels. In addition to a token encoder that is adopted in vanilla semantic-agnostic seq2seq models, they introduced a role encoder and a frame encoder which together represent semantic information produced by an off-the-shelf parser. They obtained significant gains with this semantic augmentation. Iyyer et al. [20] exploited linguistic knowledge at the syntactic level, where constituency parses are fed into their models. The authors of [21,22] did not use explicit syntactic parses, but adopted an example sentence whose underlying syntax is regarded as a syntactic exemplar. These syntax-guided approaches aimed to generate paraphrases in a controllable fashion, which is less pertinent to our work.

Another type of syntactic information is dependency trees which are concerned with how words relate to other words (constituency parses deal with how words are formed into larger units such as phrases) and contain arguably more information than constituency parses. However, adding dependency parse information in a seq2seq model to guide the generation of sentences is not trivial. Inspired by previous work employing graph convolutional networks (GCNs) to encode dependency trees in learning sentence representations, we attempt to integrate linguistic knowledge in the form of dependency parses via GCNs into the process of paraphrase generation.

## 2. Background

In this section, we provide a brief description of two component models that are necessary to understand the method proposed in this work.

### 2.1. Sequence-to-Sequence Models and Attention

NMT is typically conducted by building a neural network that takes a source sentence as input and generates its corresponding sentence in the target language. The network is made up of two component networks, namely the encoder and the decoder. An encoder–decoder network is also called a seq2seq model [23].

Formally, a source sentence $X=(x_{1},\ldots ,x_{J})$ is fed into the encoder to give a fixed-length hidden vector, presumably encoding the “meaning” of the sentence. This hidden vector is a continuous representation often referred to as the context vector. It is the last hidden state in the case of the encoder being a recurrent neural network (RNN), which is the most widely used network architecture. Then, the target sentence $Y=(y_{1},\ldots ,y_{I})$ is produced one symbol at a time, conditioning on the context vector provided by the encoder, where *I* is not necessarily equal to *J*. Essentially, the decoder is a conditional language model: given the context vector *c* and previously predicted tokens $y_{1:i-1}$, the decoder is trained to generate the next token $y_{i}$. In other words, the decoder models a distribution over possible translation sentences that can decompose into a series of individual conditionals:

$$p\left(Y\right)=\prod_{i=1}^{I}p\left(y_{i}|y_{1:i-1},c\right)$$

Each conditional probability can be defined as

$$p\left(y_{i}|y_{1:i-1},c\right)=g\left(y_{i-1},s_{i},c\right)$$

where *g* is a nonlinear function and $s_{i}$ is the hidden state for step *i* in RNN.

It is hard for the encoder to learn a fixed dimensional vector that contains the entire “meaning” of the input sentence, whose length may vary wildly. This is where the *attention* [24] mechanism comes into play. In their model, the context vector varies with time *i*, hence the probability of $y_{i}$ is conditioned on a different context vector $c_{i}$ for each prediction.

$$p\left(y_{i}|y_{1:i-1},c\right)=g\left(y_{i-1},s_{i},c_{i}\right)$$

where

$$s_{i}=f\left(s_{i-1},y_{i-1},c_{i}\right)$$

The context vector $c_{i}$ is a weighted sum of all encoder hidden states, where each weight $\alpha_{ij}$ is the amount of “attention” paid to the corresponding encoder state $h_{j}$

$$c_{i}=\sum_{j=1}^{J}\alpha_{ij}h_{j}$$

where each weight $\alpha_{ij}$ is a normalized (over all steps) attention “energy” $e_{ij}$

$$\alpha_{ij}=\frac{\exp(e_{ij})}{\sum_{k=1}^{J}\exp\left(e_{ik}\right)}$$

where each attention energy is calculated with some function *a* (such as another linear layer) using the last hidden state $s_{i-1}$ and that particular encoder output $h_{j}$:

$$e_{ij}=a\left(s_{i-1},h_{j}\right)$$

### 2.2. Graph Convolutional Networks

For a given graph G=(V,E), V is a set of nodes (vertices) and E is a set of edges (connections). Assume there are *n* nodes, each associated with a feature vector of dimensionality *d*. As we are concerned with modeling sentences, the feature vectors are simply word embeddings. Then, all node features could be represented by a matrix $X\in R^{d\times n}$. For the *i*th node *u*, the corresponding feature vector $x_{u}\in R^{d}$ is column *i* of matrix *X*. A GCN network takes two representations as input:

- node representation: feature descriptions for all nodes summarized as the feature matrix *X*; and
- graph structure representation: the topology of the graph, which is typically represented in the form of an adjacency matrix *A*.

In the first layer of a multi-layer GCN network, the node representations are computed as:

(1) $$\begin{array}{c} h_{v}=f(\sum_{u\in N(v)}Wx_{u}+b) \end{array}$$

where $W\in R^{d\times d}$ is a weight matrix and $b\in R^{d}$ is a bias vector. *W* is called filter or kernel in convolution networks. Essentially, *W* and b together constitute a linear neural network. *f* denotes an activation function such as ReLU. N(v) stands for the set of neighbor nodes of *v*, which could be obtained via the adjacency matrix *A*.

Through one GCN layer, node representations can be updated by aggregating information from immediate neighbors. By stacking GCN layers, we can allow information to propagate through multiple hops. Computation of node representations becomes recursive; for the *k*th layer, we have:

(2) $$\begin{array}{c} h_{v}^{\left(k\right)}=f(\sum_{u\in N(v)}W^{\left(k\right)}h_{u}^{\left(k-1\right)}+b^{\left(k\right)}) \end{array}$$

for the first layer k=1 and $h_{u}^{\left(k-1\right)}=h_{u}^{\left(0\right)}=x_{u}$.

#### Syntactic GCNs

Dependency trees could be formulated as directed and labeled graphs. Marcheggiani and Titov [25] generalized GCNs to take directionality and labels into account. For each node *v*, there are three possible edge directions: incoming edges (e.g., u→v), outgoing edges (e.g., v→u) and self-loop (v→v). By making weight matrices and bias vectors in a GCN network label-specific, we have:

(3) $$\begin{array}{c} h_{v}^{\left(k\right)}=f(\sum_{u\in N(v)}W_{\mathrm{lab}(u,v)}^{\left(k\right)}h_{u}^{\left(k-1\right)}+b_{\mathrm{lab}(u,v)}^{\left(k\right)}) \end{array}$$

where lab(u,v) determines the weight matrix corresponding to each direction and label combination.

Syntactic GCNs exploit the concept of gating for the graph edges. By modulating the contribution of individual edges, it allows the model to differentiate between information flowing along each edge: some edges could contain information more pertinent to the task, while others could possibly represent erroneous predicted syntactic structure. Formally, a scalar gate is calculated for each edge as follows:

(4) $$\begin{array}{c} g_{u,v}^{\left(k\right)}=\sigma (h_{u}^{\left(k-1\right)}{\hat{w}}_{\mathrm{lab}(u,v)}^{\left(k\right)}+{\hat{b}}_{\mathrm{lab}(u,v)}^{\left(k\right)}) \end{array}$$

where σ is the logistic sigmoid function and ${\hat{w}}_{\mathrm{lab}(u,v)}^{\left(k\right)}\in R^{d}$ and ${\hat{b}}_{\mathrm{lab}(u,v)}^{\left(k\right)}\in R$ are training parameters for the gate. Putting it all together, the computation for Syntactic GCNs becomes:

(5) $$\begin{array}{c} h_{v}^{\left(k\right)}=f(\sum_{u\in N(v)}g_{u,v}^{\left(k\right)}(W_{\mathrm{lab}(u,v)}^{\left(k\right)}h_{u}^{\left(k-1\right)}+b_{\mathrm{lab}(u,v)}^{\left(k\right)})) \end{array}$$

## 3. Related Work

### 3.1. Linguistic Aware Methods for Paraphrase Generation

Various researchers exploit linguistic knowledge in a paraphrase generation model, be it lexical, syntactic, or semantic knowledge. Huang et al. [15] made use of information from an off-the-shelf dictionary. Specifically, they extracted appropriate word-level and phrase-level paraphrase pairs from the PPDB database, while taking the original sentence into account. Instead of naively replacing words in the source sentence with their counterpart in the paraphrase pairs, they used these paraphrase pairs to construct edit vectors. Edit vectors are responsible for deletion and insertion operations in paraphrase generation.

Lin et al. [16] also employed an off-the-shelf dictionary containing word-level paraphrase pairs(synonyms). In addition to using these synonym pairs to guide the decision on whether to generate a new word or replace it with a synonym as in [15], they integrated information of word location with a positional encoding layer in Transformer. An interesting part of their work is that they formulated the locating of synonym candidates as a synonym labeling task, which is first trained independently. Then, the synonym labeler and the paraphrase generator are trained simultaneously to perform multi-task learning, where the two tasks share a common encoder.

Ma et al. [26] proposed to interact with distributed word representations instead of the corresponding words per se. The hidden vector from a neural network is used as a query. A dictionary is constructed, which is basically a word embedding lookup table with its keys and values swapped. When the network is making predictions, a matching score is computed between the query and the embedding keys, the key obtaining the highest score is retrieved, and the associated value (word) is returned. Hence, the model generates the words in a retrieval style. However, they did not experiment with standard paraphrase generation but two closely related tasks: text simplification and short text abstractive summarization.

Another line of research takes advantage of linguistic knowledge at the syntactic level. Iyyer et al. [20] proposed to incorporate linearized constituency parses from paraphrase pairs to control the syntax of generated paraphrases. In this way, their model learns the syntactic transformations that naturally occur in paraphrase data. Samples produced by the method could fool traditional paraphrase detection models due to syntactic variations. Thus, such adversarial examples could be used to augment the training data for paraphrase detection models to improve their robustness.

Chen et al. [21] adopted a syntactic exemplar sentence as an alternative to the explicit target syntactic form used in [20]. The syntactic exemplar is composed such that its semantics deviate from the input sentence to be paraphrased. The generated paraphrase following the semantics of the first sentence and syntax of the second sentence(syntactic exemplar).

Similarly, Kumar et al. [22] also used a syntactic exemplar to conduct paraphrase generation with controlled syntax. Their model utilizes full exemplar syntactic tree information and is capable of regulating the granularity level of syntactic control.

Wang et al. [18] added semantic information in their paraphrase generation model. PropBank style semantic information is obtained through an off-the-shelf frame-semantic parser. Each token in an input sentence is associated with a frame and a role label, resulting in three input channels which are fed into three separate encoders. The results are then aggregated with a linear layer and fed into the decoder to generate a paraphrase.

### 3.2. GCN for NLP

Graph Convolutional Networks has been applied in a range of NLP problems. Here, we briefly overview some relevant works employing syntactic GCNs. Marcheggiani and Titov [25] proposed to adopt syntactic GCNs for semantic role labeling. Word embeddings are first fed into a bidirectional LSTM network to obtain hidden word representations. Then, these hidden word vectors are re-encoded by the following GCN network. Finally, the hidden vectors are passed to a classifier to predict a semantic label.

Vashishth et al. [27] employed syntactic GCNs to learn word embeddings. They generalized the continuous-bag-of-words (CBOW) [28] model by substituting the sequential context (words within a fixed window size) with a syntactic context (neighbor words in a dependency tree). By exploiting syntax information in selecting and weighting context words, their approach produced more meaningful word representations.

Bastings et al. [29] proposed to use syntactic GCNs for English–German and English–Czech machine translation. They fixed the decoder as a recurrent network and explored baseline models combined with GCN to compose the encoder. Specifically, the baselines they examined are a bag-of-words encoder, a recurrent encoder, and a convolutional encoder. When stacking a GCN network on each baseline encoder, significant performance gains were observed for both translation tasks.

## 4. Methods

We focus on the case where dependency information on the source side is available and hypothesize that by integrating syntax information with GCNs, the encoder would learn more meaningful sentence representations. We assume that such enriched representations, when fed into the decoder, would lead to improved quality in generated paraphrases. For RNN networks, we employ Gated Recurrent Units (GRUs). Now, we describe the encoder and the decoder, respectively.

### 4.1. Encoder

The encoder composes three modules: an embedding module, a bidirectional RNN (BiRNN) module, and a GCN module. The embedding module is essentially a lookup table that turns word indices into corresponding dense vectors. These word vectors are then processed by BiRNN networks, obtaining a sentence representation. This sentence vector is then refined by the GCN module that follows. The GCN network takes syntax information in the form of an adjacency matrix as input. The adjacency matrix for each sentence remains constant in the process of model training. Let $x_{j}$ denote the embedding vector for word *j*; hidden vectors output by the forward GRU and backward GRU at time step *j* are computed as:

$${\vec{h}}_{j}={\mathrm{GRU}}_{f}\left(x_{j},{\vec{h}}_{j-1}\right)$$

$${\overset{\leftarrow }{h}}_{j}={\mathrm{GRU}}_{b}\left(x_{j},{\overset{\leftarrow }{h}}_{j+1}\right)$$

where ${\mathrm{GRU}}_{f}$ and ${\mathrm{GRU}}_{b}$ denote the forward GRU and the backward GRU respectively. Then, the two hidden vectors are concatenated as a single vector:

$$h_{j}={\vec{h}}_{j}⊕{\overset{\leftarrow }{h}}_{j}$$

This is taken as input for the GCN module:

$${\tilde{h}}_{j}=\mathrm{GCN}\left({\left\{h_{j}\right\}}_{j=1}^{J},A\right)$$

where *A* represents the adjacency matrix determining the neighborhood of node *j*. Here, we assume the length of source sentence is *J*, input $h_{j}$ is the hidden vector for word *j* and ${\left\{h_{j}\right\}}_{j=1}^{J}$ stands for the set of hidden vectors each corresponds to a word in the source sentence.

### 4.2. Decoder

For the decoder part, we also adopt a GRU network. Let $s_{i}$ denote the hidden state vector at time step *i*. A context vector is computed by an attention module, taking the set of all encoder hidden states and the previous decoder hidden state:

$$c_{i}=\mathrm{Attention}\left({\left\{{\tilde{h}}_{j}\right\}}_{j=1}^{J},s_{i-1}\right)$$

the context vector is concatenated with the previous target word vector $y_{i-1}$ and fed into a GRU:

$$s_{i}=\mathrm{GRU}\left(s_{i-1},c_{i}⊕y_{i-1}\right)$$

Finally, these three vectors are passed through a linear layer followed by a softmax function to produce a probability for the next word:

$$p\left(y_{i}\right)=\mathrm{Softmax}\left(\mathrm{Linear}\left(s_{i}⊕c_{i}⊕y_{i-1}\right)\right)$$

**Figure 1:**
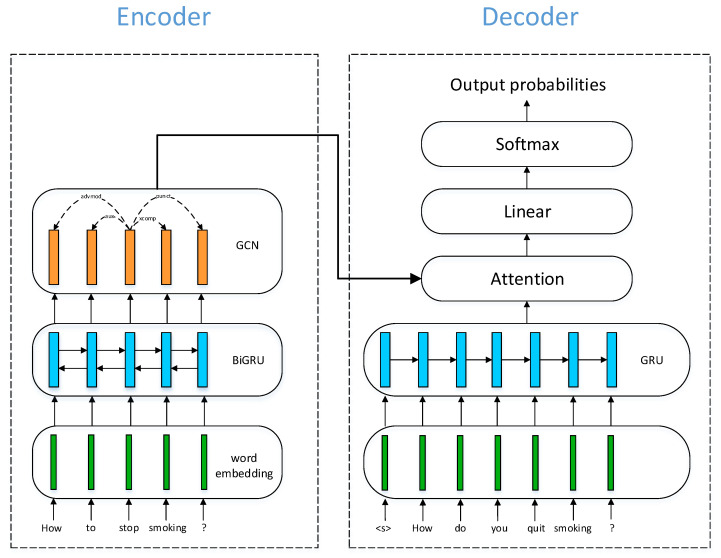
Model architecture. Vectors are depicted as colored rectangles, including word embeddings and hidden vectors learned by neural nets. Arrows denote information flow. For the GCN part, we also show the dependency relations for the example input sentence as dashed arrows. The specific dependency types are annotated on the corresponding arrows.

At inference time, we use a greedy decoder, selecting the output token with the highest probability at each time step. An overview of the model architecture is illustrated in Figure 1.

## 5. Experiments

Experiments were performed with PyTorch(version 1.4.0). Its official website is https://pytorch.org/ (accessed on 16 July 2020). We used torchtext (version 0.6.0), which is shipped with PyTorch; it provides a range of convenient text processing utilities. We employed spaCy (version 2.3.2) to perform tokenization and dependency parsing; the spaCy model we used is en-core-web-sm-2.3.1. Encoder–decoder code is based on [30]. GCNs are also implemented with PyTorch. Source code for the GCN part is available at https://github.com/chifish/SyntacticGCN (accessed on 2 March 2021).

### 5.1. Datasets

Following previous work on the task of paraphrase generation, we used two widely investigated paraphrase datasets, namely Quora and ParaNMT [14]. The Quora dataset is available at https://www.kaggle.com/c/quora-question-pairs (accessed on 11 December 2020). Released in January 2017, it is originally developed for classifying whether question pairs have the same intent. Each line in the dataset consists of question numbers(IDs), followed by the text of each question and a binary value given by human annotators indicating whether the pair is considered duplicate or not. If the label is “1”, the question pair is indeed paraphrases of each other. We further split the Quora dataset into three datasets with increasing sizes: Quora50K, Quora100K, and Quora150K, where each dataset is a subset of a larger dataset. There are actually 134K sentence pairs in Quora150K, and we keep this naming to be consistent with previous work. The minimum frequency for building vocabulary was set to 3 for these three Quora datasets.

The ParaNMT dataset was constructed by translating the Czech side of a large English–Czech parallel corpus into English using NMT. A paraphrase is formed by pairing the English translation with the corresponding reference sentence. The main purpose of creating this large paraphrase corpus is to learn sentence representations whose superiority is manifested in a semantic textual similarity task. However, it is also shown to be useful in paraphrase generation tasks. A score is associated with each paraphrase pair indicating the level of similarity. The scores are divided into five ranges. After manually checking a random sample from the highest score range, we found that a large portion reveals a remarkable level of lexical overlap between sentence pairs. We believe this phenomenon is not desirable in generated paraphrases since generally (at least lexically) divergent sentences are deemed more interesting and useful. Thus, we chose the second-highest score range (0.6–0.8) for this work. The dataset exhibits a high level of noise in various forms, so we filtered out noisy sentences. The script for this filtering step is available at https://github.com/chifish/preprocess (accessed on 29 March 2021). Finally, we obtained 2.3 million sentence pairs. When building the vocabulary with the training set, we kept those tokens that appear at least 10 times, resulting in a vocabulary size of about 50K.

Previous work truncates sentences in both datasets at the length of 15. This procedure would result in incomplete graph structures for models utilizing GCNs. Thus, it is necessary to keep whole sentences. Filtering out sentences with lengths beyond 15 would result in a substantial loss in data volume. We decided to reserve those sentences whose lengths are at most 30 for all four datasets. We randomly sampled 2K sentence pairs for development and 10K for test, respectively.

### 5.2. Training Configuration

We used the Adam optimizer [31] with an initial learning rate of 0.001. We utilized early-stopping [32] to monitor training and mitigate overfitting on training data. The model with the highest validation BLEU was saved for testing. Word embedding size was set to 256; hidden size was set to 128, and batch size was set to 128. Sentences of similar length were bucketed together to minimize padding and hence increase training efficiency. This bucketing procedure was realized through torchtext. Between layers, we applied dropout with a probability of 0.2. In experiments with GCNs, we also used the value of 0.2 for edge dropout. These dropout rates were tuned on the validation set. To mitigate the effect of randomness on model performance, we ran each model five times, each time with a random seed chosen from 42 to 46. The performance scores were averaged across five runs. Considering the wide gap between the sizes of the Quora dataset and the ParaNMT dataset, we adopted different configurations in several aspects, which we introduce below in detail.

- For the three Quora datasets, evaluation was performed on the validation set at the end of each epoch. The learning rate was halved if the validation loss did not decrease for two consecutive evaluations. The stopping criterion was the BLEU score, and the tolerance number was set to 5, so training was terminated if BLEU scores on 5 consecutive evaluations on the development set did not improve. Since the Quora datasets are relatively small, we used one layer in both encoder (bidirectional) and decoder.
- For the ParaNMT dataset, we evaluated the model every 2000 training steps. The criterion adopted for early-stopping was BLEU, and the tolerance number was set to 10, which is the minimum to cover an entire epoch. The layer depths of both encoder and decoder were set to 2, where the encoder is again bidirectional.

### 5.3. Evaluation Metrics

As opposed to NMT, there is no consensus on what metrics should be used for evaluating paraphrase generation models. As a result, measurements adopted by different works vary. In this work, we report model performance on multiple metrics to provide a more comprehensive understanding.

- BLEU [33] counts the overlap of sentence fragments in reference translations and the candidate translation output by NMT systems. Assume there are two reference translations and a candidate translation. For each word type in the candidate, the number of times it occurs in both reference sentences are computed, and the maximum of these two counts is taken as an upper bound. Then, the total count of each candidate word is clipped by this upper bound. Next, these clipped counts are summed up. Finally, this sum is divided by the total (unclipped) number of candidate words. This is the case for single words or unigrams. The score for other n-grams is computed similarly. Typically, n ranges from 1 to 4.
- METEOR [34] calculates unigram matching in a generalized fashion. For unigrams, BLEU only takes surface form into account. In contrast, METEOR also matches stem, synonym, and paraphrase between candidate and reference.
- ROUGE [35] is commonly employed in the evaluation of automatically generated summaries. However, it is also used to assess paraphrases. There are four types of ROUGE, among which ROUGE-n that deals with n-grams is most pertinent to paraphrase evaluation. Assume there are two reference sentences. For each n-gram in each of the two reference sentences, the number of times the n-gram occurs in the candidate is calculated. The maximum of these two counts is kept. Then, the maximum counts for each n-gram are summed, which is then divided by the total number of counts of n-grams in the references. We also report results with another type of ROUGE: ROUGE-L. It operates on longest common subsequence. The ROUGE scores presented in this work are F-measure values, which is a trade-off between precision and recall.

## 6. Results and Discussion

Now, we report the experiment results on all four datasets.

### 6.1. Prediction Scores

We evaluated paraphrases generated by our models given test set source sentences as input. Scores were computed between prediction sentence and ground-truth pairs on the corpus level. We present the average scores across five random runs for each model and dataset combination. The set of random seeds was repeated for each combination. Standard deviations are shown in parentheses that follow. Significance test was performed against RNN.

Performance detailed in Table 1 shows that our GCN-enhanced models almost uniformly outperform the RNN baseline models at a significance level of p<0.01. The only exception is the BLEU score for Quora150K.

### 6.2. Models for Comparison

We also compared our models with previous work on the Quora150K dataset. Since previous work truncated sentences beyond length 15, for a fairer comparison, we filtered out test sentences with length over 15 (reducing the test set from 10K to 8.6K) and calculated METEOR and BLEU scores for our models on this smaller dataset. The results are illustrated in Table 2.

Previous work for comparison are as follows:

- Residual LSTM [11] is the first seq2seq model proposed for paraphrase generation.
- FSET [36] is a retrieval-based method for paraphrase generation that paraphrases an input sequence by editing it using an edit vector which is composed of the extracted relations between a retrieved sentence pair.
- KEPN [16] employs an off-the-shelf dictionary containing word-level paraphrase pairs(synonyms). In addition to using these synonym pairs to guide the decision on whether to generate a new word or replace it with a synonym, it integrates information of word location with a positional encoding layer in Transformer.
- VAE-SVG-eq [37] is a variational autoencoder (VAE) based on neural networks that conditions both the encoder and decoder of VAE on the input sentence.

As can be seen in the table above, our models produce higher scores in METEOR but lower scores in the BLEU metrics. These two metrics differ in that METEOR takes synonym information into account, so that when a word in the generated sentence is different from a word in the reference sentence in surface form but they are synonyms to each other, they would be still deemed a match by METEOR but not by BLEU(see Section 5.3 for detailed descriptions of these two metrics). As a result, these synonyms would contribute to the METEOR score. We hypothesize that GCN-enhanced models are better at capturing synonym information, yet the reason behind this phenomenon needs further study.

### 6.3. Effect of Sentence Length

**Figure 2:**
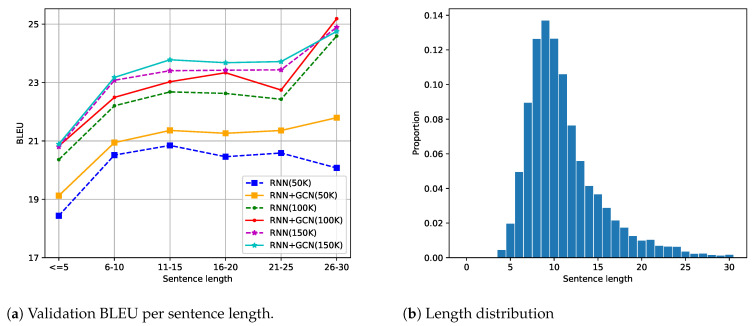
Effect of sentence length for Quora datasets. (**a**) The relationship between BLEU score and sentence length on three Quora datasets. Dataset sizes are given in parentheses. (**b**) Distribution of sentence length for Quora50K, where the patterns for the two larger Quora datasets are similar.

We expect that GCN networks would show a larger advantage for longer sentences since they contain long-distance syntactic dependencies that would be challenging for RNNs to model but can be easily captured via syntactic connections encoded by GCNs. To verify this hypothesis, we split the sentences predicted by models trained on each Quora dataset into six buckets and compute BLEU scores for each separate bucket. Figure 2a shows that GCNs do outperform syntax-agnostic RNN models by a larger margin for longer sentences on the Quora50K dataset. For Quora100K, the advantage of GCN models is relatively uniform. For Quora150K, the distinction between two models is marginal when sentence length goes to two extremes. One explanation is data sparsity for those two length ranges. As shown in Figure 2b, most sentences fall into two buckets (6–10 and 11–15), which account for 53% and 31%, respectively, for all three datasets. Sentences that contain no more than five words take up a share of only 2.4%, and sentences that fall into the longest bucket are most scarce, with a low proportion of less than 1%. We also draw the standard deviation of each performance score; the variation for buckets at both ends is indeed much higher.

### 6.4. Discussion

Splitting the Quora dataset into different sizes allows us to study another dimension of GCNs: the effect of dataset size on model performance. A close look at Figure 2a tells us that, as the dataset grows, the performance gains of GCN models over baseline RNNs declines. This aspect can also be deduced from the results in Table 1, where smaller datasets show a higher level of significance difference between the two model architectures. To be specific, the *p*-values (lower values signify higher significance levels) for the three datasets are in the order of magnitude of $10^{-5}$, $10^{-4}$, and $10^{-2}$, respectively. We hypothesize that, when given sufficient data, RNNs could model some aspects of syntactic structure; hence the advantage of GCN-based models gradually disappears. This is presumably the case for the Quora dataset, which mainly contains questions with simple syntactic patterns. Bastings et al. [29] considered datasets of differing sizes. Specifically, for the English–German machine translation task, they investigated two dataset sizes: 226K and 4.5M. The BLEU${}_{1}$ score gain they monitored dropped from 2.3 to 1.6, and the gain in BLEU${}_{4}$ dropped from 1.2 to 0.6.

We expected GCN models to outperform RNNs by a narrower margin as the dataset size rises to the order of millions. Surprisingly, the BLEU score margin for GCN models increases to nearly one point on the ParaNMT dataset with over two million training samples. This again testifies the efficacy of GCN-based models for encoding syntax information.

## 7. Conclusions

In this paper, we propose to encode syntax information for paraphrase generation via graph convolutional networks. GCNs allow us to encode sentences via dependency trees. By stacking GCNs on top of recurrent networks on the encoder side of our paraphrasing model, we achieved significantly better results on four paraphrase datasets of varying sizes and genres. We hypothesize that dependency relations contain rich syntactic information that is valuable in learning sentence representation. Besides, convolutional operations in GCNs have complementary power to recurrent connections in RNNs. Through analyzing the experiment results using multiple evaluation metrics on a range of paraphrase datasets, we demonstrated the efficacy of our approach. We also studied the effects sentence length and size of training data have on model performance and found that recurrent networks enhanced by GCNs are more effective for smaller datasets when the dependency patterns are less diverse. However, for sentences that are either too short or too long, it becomes challenging for GCNs to learn more meaningful representations. This is mainly due to data sparsity. We plan to apply GCNs in other text generation tasks such as question answering and dialog systems in future work.

## Figures and Tables

**Table 1:** Test performance in five metrics. We use the dagger symbol (“†”) to denote significance levels, where “†” indicates significantly better than RNN (p<0.05) and “††” indicates significantly better than RNN (p<0.01).

Dataset	Model	BLEU	METEOR	ROUGE-1	ROUGE-2	ROUGE-L
Quora50K	RNN	20.57 (±0.12)	47.3 (±0.14)	48.63 (±0.15)	28.12 (±0.15)	46.84 (±0.13)
	RNN + GCN	21.09 (±0.07) ^††^	48.41 (±0.1) ^††^	49.84 (±0.1) ^††^	28.93 (±0.09) ^††^	48.0 (±0.1) ^††^
Quora100K	RNN	22.38 (±0.08)	49.85 (±0.07)	51.64 (±0.12)	30.57 (±0.08)	49.56 (±0.09)
	RNN + GCN	22.74 (±0.1) ^††^	50.59 (±0.1) ^††^	52.72 (±0.09) ^††^	31.42 (±0.09) ^††^	50.67 (±0.11) ^††^
Quora150K	RNN	23.19 (±0.06)	50.89 (±0.05)	52.86 (±0.07)	31.71 (±0.02)	50.7 (±0.06)
	RNN + GCN	23.4 (±0.13) ^†^	51.4 (±0.19) ^††^	53.68 (±0.11) ^††^	32.31 (±0.16) ^††^	51.53 (±0.11) ^††^
ParaNMT	RNN	16.15 (±0.37)	43.32 (±0.49)	46.66 (±0.34)	22.58 (±0.35)	43.48 (±0.33)
	RNN + GCN	17.09 (±0.15) ^††^	44.52 (±0.27) ^††^	47.65 (±0.19) ^††^	23.5 (±0.18) ^††^	44.49 (±0.2) ^††^

**Table 2:** Comparison with previous work on Quora150K. BLEU-2 and BLEU-4 scores of the method marked with a “*” are taken from Kazemnejad et al. [36]. The other scores in the first four rows are taken from the corresponding papers.

Model	METEOR	BLEU	BLEU-1	BLEU-2	BLEU-3	BLEU-4
Residual LSTM [11] *	28.9	27.4	-	38.52	-	24.56
FSET [36]	38.57	-	-	51.03	-	33.46
KEPN [16]	30.4	29.2	-	-	-	-
VAE-SVG-eq [37]	33.6	38.3	-	-	-	-
RNN(ours)	50.88	23.14	49.0	27.78	17.72	11.89
RNN+GCN(ours)	51.39	23.34	49.04	28.13	17.95	11.99

## Data Availability

The datasets investigated in this work are publicly available at https://www.kaggle.com/c/quora-question-pairs (accessed on 11 December 2020) and https://drive.google.com/file/d/1rbF3daJjCsa1-fu2GANeJd2FBXos1ugD/view (accessed on 18 May 2019).

## Abbreviations

The following abbreviations are used in this manuscript:

NLP	Natural Language Processing
GCN	Graph Convolutional Network
RNN	Recurrent Neural Network
BiRNN	Bidirectional Recurrent Neural Network
GRU	Gated Recurrent Unit
LSTM	Long Short-Term Memory
NMT	Neural Machine Translation
CBOW	continuous-bag-of-words
